# RAGE is essential for oncogenic KRAS-mediated hypoxic signaling in pancreatic cancer

**DOI:** 10.1038/cddis.2014.445

**Published:** 2014-10-23

**Authors:** R Kang, W Hou, Q Zhang, R Chen, Y J Lee, D L Bartlett, M T Lotze, D Tang, H J Zeh

**Affiliations:** 1 Division of Gastrointestinal Surgical Oncology, Department of Surgery, University of Pittsburgh Cancer Institute, University of Pittsburgh, Pittsburgh, PA 15219, USA

## Abstract

A hypoxic tumor microenvironment is characteristic of many cancer types, including one of the most lethal, pancreatic cancer. We recently demonstrated that the receptor for advanced glycation end products (RAGE) has an important role in promoting the development of pancreatic cancer and attenuating the response to chemotherapy. We now demonstrate that binding of RAGE to oncogenic KRAS facilitates hypoxia-inducible factor 1 (HIF1)*α* activation and promotes pancreatic tumor growth under hypoxic conditions. Hypoxia induces NF-*κ*B-dependent and HIF1*α*-independent RAGE expression in pancreatic tumor cells. Moreover, the interaction between RAGE and mutant KRAS increases under hypoxia, which in turn sustains KRAS signaling pathways (RAF-MEK-ERK and PI3K-AKT), facilitating stabilization and transcriptional activity of HIF1*α*. Knock down of RAGE *in vitro* inhibits KRAS signaling, promotes HIF1*α* degradation, and increases hypoxia-induced pancreatic tumor cell death. RAGE-deficient mice have impaired oncogenic KRAS-driven pancreatic tumor growth with significant downregulation of the HIF1*α* signaling pathway. Our results provide a novel mechanistic link between NF-*κ*B, KRAS, and HIF1*α*, three potent molecular pathways in the cellular response to hypoxia during pancreatic tumor development and suggest alternatives for preventive and therapeutic strategies.

Pancreatic cancer is the fourth leading cause of cancer mortality in the USA.^1^ The high mortality rate is due to the high incidence of metastatic disease at initial diagnosis, aggressive clinical course, and failure of systemic therapies. Over the past decade, numerous trials have been conducted to improve the outcomes in patients with metastatic disease by combination therapies employing gemcitabine as a foundation.^2^ Despite a modest improvement in progression-free survival in combination with other drugs, the longer disease-free survival has not translated into any advantage in the overall survival.^3^ New therapeutic strategies that specifically target cancer-specific pathways are required for the successful treatment and prevention of this disease. The small guanosine triphosphatase KRAS is frequently mutated in 98% of pancreatic cancers, making it an ideal target for therapeutic intervention.^4, 5, 6^ Attempts to target this pathway have largely been unsuccessful. A better understanding of the molecular pathways involved in KRAS-driven development and progression of pancreatic cancer could potentially lead to improved targeted therapies.^7^

Evidence from experimental and clinical studies increasingly points to an important role for hypoxia in the pathogenesis of solid cancers,^8^ including pancreatic cancer.^9, 10, 11, 12, 13, 14^ Hypoxia-inducible factor 1 (HIF1) is a master transcription factor that regulates the oxygen supply balance and demand in response to intratumoral hypoxia and aberrantly, with oncogenic mutations.^15^ HIF1 is a heterodimer composed of an *α*-subunit (HIF1*α*) and a *β*-subunit (HIF1*β*). Its activity primarily depends on the stability and modification of HIF1*α*.^15^ Under normoxic conditions, HIF1*α* is unstable and undergoes immediate proteasomal degradation. Under hypoxic conditions, HIF1*α* is stabilized and induces the transcription of several genes involved in the development of inflammation, anaerobic metabolism, metastasis, and drug resistance.^8,15^

The receptor for advanced glycation end products (RAGE, also termed AGER) is a transmembrane receptor of the immunoglobulin super family encoded within the Class III region of the major histocompatibility complex. RAGE activation is responsible for enhancing inflammation and has been implicated in several chronic diseases including diabetes, neurodegenerative disorders, and cancer.^16,17^ RAGE is expressed by malignant cells as well as other cells within the tumor microenvironment including leukocytes, endothelial cells, and fibroblasts. We recently demonstrated that RAGE has a unique role in pancreatic tumorigenesis and drug resistance.^18, 19, 20, 21, 22^ Knock down or knock out of RAGE *in vitro* or *in vivo* attenuates oncogenic KRAS-driven pancreatic tumor progression^19^ and reverses the resistance to multiple drugs.^20,21^ We demonstrated that the mechanism by which this occurs is partly involved in inflammatory response-associated metabolic changes^19,22,23^ and cell death-associated autophagy.^20,21,24,25^ Thus, targeting RAGE represents a novel approach for pancreatic cancer therapy.

Using murine studies as well as molecular, and cellular studies *in vitro*, we demonstrate here that binding of RAGE to mutant KRAS is a direct, positive regulator of HIF1*α*-dependent hypoxia signaling in pancreatic tumor development. These findings further refine our understanding of how RAGE promotes the development of pancreatic cancer and offer a novel mechanism by which an inflammatory gene can promote cell survival through hypoxic pathways.

## Results

### Hypoxia increases RAGE expression through the NF-*κ*B pathway in pancreatic tumor cells

**Figure 1:**
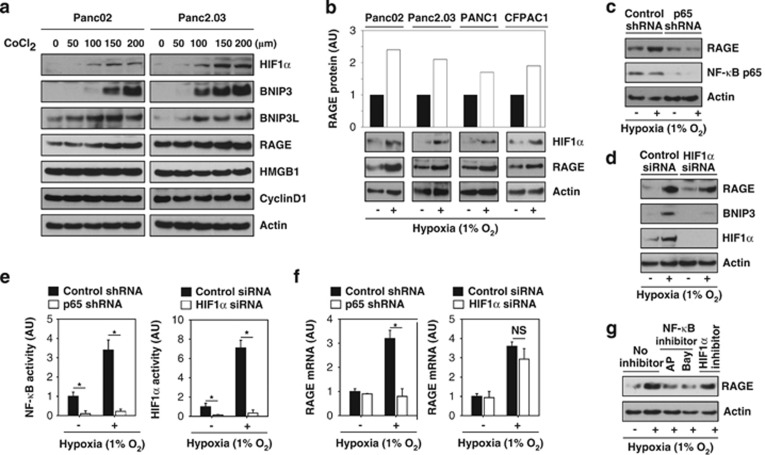
Hypoxia increases RAGE expression in an NF-*κ*B-dependent and HIF1*α*-independent manner in pancreatic tumor cells. (**a** and **b**) Indicated pancreatic tumor cell lines were treated with CoCl_2_ (**a**) or 1% O_2_ (**b**) for 24 h. Western blot analyzed expression of RAGE, HIF1*α*, and other indicated proteins. (**c** and **d**) Panc02 cells were transfected with control shRNA (**c**) p65 shRNA (**c**) control siRNA (**d**) and HIF1*α* siRNA (**d**) for 48 h, and then treated with 1% O_2_ for 24 h. The indicated protein levels were analyzed by western blot. (**e** and **f**) In parallel, the transcriptional activity of NF-*κ*B and HIF1*α* (**e**) and RAGE mRNA level (**f**) were assayed (*n*=3, *P<0.05). (**g**) Panc02 cells were treated with 1% O_2_ with or without NF-*κ*B inhibitor (ammonium pyrrolidinedithiocarbamate (AP, 100 *μ*M)) and Bay 11–7082 (Bay, 10 *μ*M) and HIF1*α* inhibitor (methyl 3-((2-(4-(2-adamantyl)phenoxy)acetyl)amino)-4-hydroxybenzoate, 10 *μ*M) for 24 h. The protein level of RAGE was analyzed by western blot. AU=arbitrary unit

To evaluate whether RAGE is induced by hypoxia, we first treated two individual pancreatic tumor cell lines (mouse Panc02 and human Panc2.03) with cobalt chloride 2 (CoCl_2_), a widely used chemical inducer of hypoxia.^26^ As expected,^27^ CoCl_2_ prevented HIF1*α* degradation and increased HIF1*α* expression. Similarly, the expression of the HIF1a target genes BCL2/adenovirus E1B 19 kDa interacting protein 3 (BNIP3) and BNIP3L/NIX were significantly increased following CoCl_2_ treatment in pancreatic tumor cells (Figure 1a).^26^ In contrast, non-target genes (e.g., high mobility group box 1 (HMGB1) and cyclinD1) did not change (Figure 1a). Interestingly, the expression of RAGE was increased following CoCl_2_ (Figure 1a) or hypoxic (1% O_2_) treatment (Figure 1b) in multiple pancreatic tumor cell lines, suggesting a potential role of RAGE in the response to hypoxia. Several transcription factors, including NF-*κ*B and HIF1*α*, are required for upregulated RAGE expression in response to stress in cancer and non-cancer cells.^20,28,29^ To explore whether NF-*κ*B and HIF1*α* are involved in hypoxia-induced RAGE expression in pancreatic tumor cells, we knocked down NF-*κ*B p65 and HIF1*α* in Panc02 cells by specific shRNA (Figure 1c) and siRNA (Figure 1d), respectively. Suppression of NF-*κ*B p65 and HIF1*α* expression significantly limited hypoxia-induced transcriptional activity of both genes (Figure 1e). As expected, suppression of HIF1*α* inhibited hypoxia-induced BNIP3 expression (Figure 1d). However, suppression of NF-*κ*B p65, but not HIF1*α*, inhibited hypoxia-induced expression of RAGE protein (Figures 1c and d) and mRNA expression (Figure 1f). Moreover, the NF-*κ*B inhibitor (e.g., ammonium pyrrolidinedithiocarbamate (AP) and Bay 11-7082 (Bay)), but not the HIF1*α* inhibitor (e.g., methyl 3-((2-(4-(2-adamantyl)phenoxy)acetyl)amino)-4-hydroxybenzoate), inhibited hypoxia-induced RAGE protein (Figure 1g) and mRNA (data not shown) expression. These findings suggest that hypoxia-induced RAGE expression is NF-*κ*B-dependent and HIF1*α*-independent.

### NF-*κ*B-mediated RAGE expression increases stabilization and transactivation of HIF1*α* under hypoxic conditions in pancreatic tumor cells

**Figure 2:**
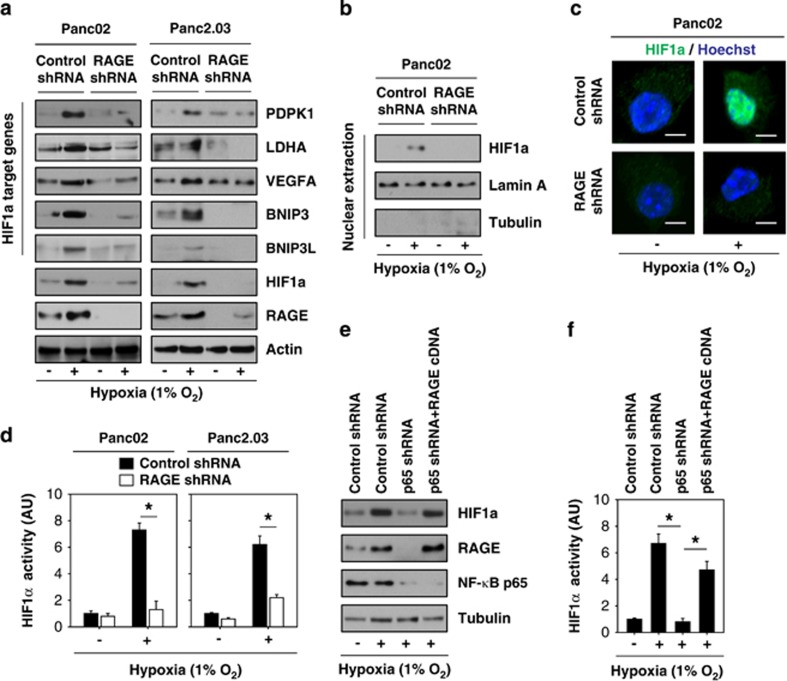
NF-*κ*B-mediated RAGE expression positively regulates HIF1*α* signaling under hypoxia in pancreatic tumor cells. (**a**) Indicated pancreatic tumor cell lines were transfected with control shRNA and RAGE shRNA for 48 h and then treated with 1% O_2_ for 24 h. Western blot was used to analyze the expression of RAGE, HIF1*α*, and HIF1*α*-target proteins. (**b**–**d**) In parallel, nuclear HIF1*α* level (**b** and **c**) and the transcriptional activity of HIF1*α* (**d**) were assayed (*n*=3, *P<0.05). Bar=10 *μ*m. (**e** and **f**) Panc02 cells were transfected with indicated shRNA and cDNA for 48 h and then treated with 1% O_2_ for 24 h. The indicated protein levels were analyzed by western blot (**e**). In parallel, the transcriptional activity of HIF1*α* (**f**) was analyzed (*n*=3, *P<0.05). AU=arbitrary unit

Given that hypoxia increases RAGE expression independent of HIF1*α* we next asked whether RAGE has a role in the regulation of HIF1*α*'s stabilization and transactivation. RAGE was knocked down by specific shRNA in Panc02 and Panc2.03 cells (Figure 2a). In contrast to the control shRNA group, the knockdown of RAGE significantly inhibited hypoxia-induced HIF1*α* expression (Figure 2a), HIF1*α* nuclear translocation (Figures 2b and c), and transactivation of HIF1*α* (Figure 2d). Moreover, knockdown of RAGE also inhibited hypoxia-induced protein expression of HIF1*α* target genes such as 3-phosphoinositide-dependent protein kinase 1 (PDPK1), lactate dehydrogenase A (LDHA), vascular endothelial growth factor A (VEGFA), BNIP3, and BNIP3L (Figure 2a). Because NF-*κ*B promotes RAGE expression in the setting of hypoxia, we next examined whether knockdown of NF-*κ*B p65 by shRNA could affect stabilization and transactivation of HIF1*α*. Consistent with a previous study, the knock down of NF-*κ*B p65 inhibits hypoxia-induced HIF1*α* expression (Figure 2e) and transactivation (Figure 2f). Of note, forced overexpression of RAGE by gene transfection restores hypoxia-induced HIF1*α* expression (Figure 2e) and transactivation (Figure 2f) in NF-*κ*B p65-knockdown Panc02 cells. Such results suggest that NF-*κ*B-mediated RAGE expression is a positive regulator of the HIF1*α* signaling pathway, regulating its stabilization and transactivation.

### RAGE-mediated KRAS pathway activation contributes to hypoxia-induced HIF1*α* activity in pancreatic tumor cells

**Figure 3:**
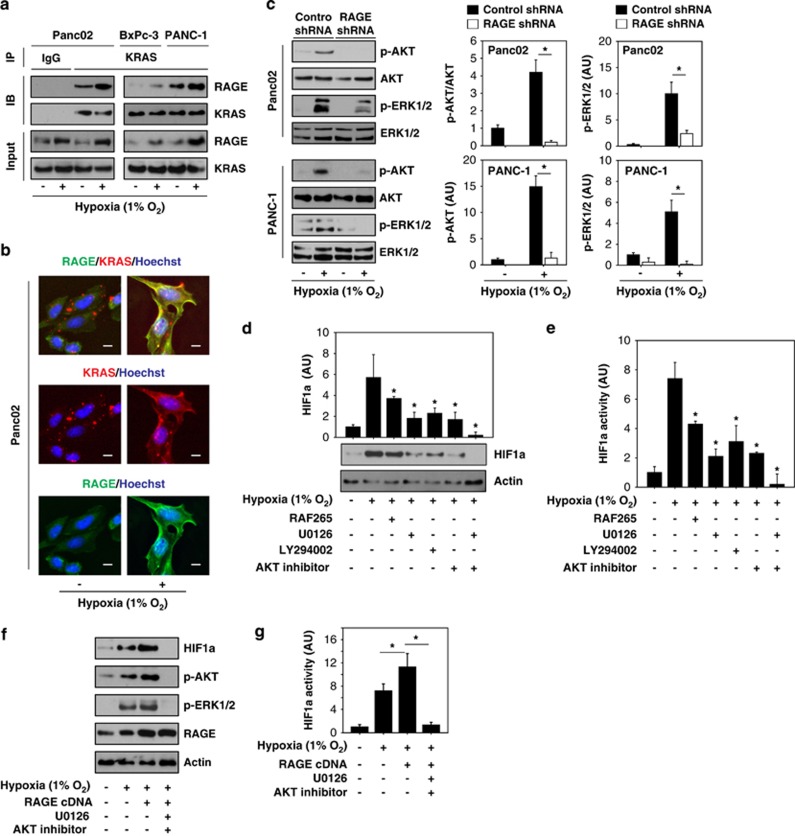
RAGE-mediated KRAS pathway activation promotes hypoxia-induced HIF1*α* expression and activity in pancreatic tumor cells. (**a** and **b**) Indicated pancreatic cancer cells were treated with 1% O_2_ for 24 h. The interaction between RAGE and KRAS were assayed by immunoprecipitation (**a**) and image analysis (**b**). Bar=10 *μ*m. (**c**) Panc02 and PANC-1 cells were transfected with control shRNA and RAGE shRNA for 48 h, and then treated with 1% O_2_ for 24 h. Western blot was used to analyze the expression of p-AKT, AKT, p-ERK1/2, and ERK1/2. Relative p-AKT and p-ERK1/2 levels were quantified (*n*=3, *P<0.05). (**d** and **e**) Panc02 cells were treated with 1% O_2_ for 24 h with or without potential RAF inhibitor (e.g., RAF265, 1 *μ*M), MEK inhibitor (e.g., U0126, 10 *μ*M), PI3K inhibitor (e.g., LY294002, 10 *μ*M), and AKT inhibitor (e.g., 1,3-Dihydro-1-(1-((4-(6-phenyl-1H-imidazo(4,5-g)quinoxalin-7-yl)phenyl)methyl)-4-piperidinyl)-2H-benzimidazol-2-one trifluoroacetate salt hydrate, 25 *μ*M). The protein expression (**d**) and transcriptional activity (**e**) of HIF1*α* were assayed (*n*=3, *P<0.05 *versus* hypoxia group). (**f** and **g**) Normal or RAGE-overexpressed Panc02 cells were treated with 1% O_2_ for 24 h with or without MEK inhibitor (e.g., U0126, 10 *μ*M) and AKT inhibitor (e.g., 1,3-Dihydro-1-(1-((4-(6-phenyl-1H-imidazo(4,5-g)quinoxalin-7-yl)phenyl)methyl)-4-piperidinyl)-2H-benzimidazol-2-one trifluoroacetate salt hydrate, 25 *μ*M). The indicated protein expression (**d**) and transcriptional activity of HIF1*α* (**e**) were assayed (*n*=3, *P<0.05). AU=arbitrary unit

We next evaluated potential mechanisms by which RAGE could enhance HIF1*α*- signaling under hypoxia. Our previous work demonstrated that RAGE has a critical role in the regulation of KRAS-driven pancreatic tumorigenesis.^19^ We now examined whether RAGE and KRAS directly interacted with each other. Immunoprecipitation (Figure 3a) and image analysis (Figure 3b) uncovered an interaction between RAGE and KRAS in KRAS-mutant Panc02 cells. Furthermore, this interaction increased under hypoxic conditions (Figures 3a and b), suggesting a direct role of RAGE in the regulation of KRAS signaling. The most frequent mutation in pancreatic cancer is the constitutively active KRAS^G12D^ allele. Hypoxia-induced interaction between RAGE and KRAS was significantly increased in KRAS^G12D^ mutant PANC-1 cells compared with KRAS wild-type BxPc-3 cells (Figure 3a), suggesting that mutant KRAS preferentially associates with RAGE. RAF-MEK-ERK and PI3K-AKT are the two main effector pathways of KRAS.^4^ To determine the effects of RAGE on the activation of these effector pathways, we monitored the level of hypoxia-induced phospho-Akt (p-AKT) and phospho-ERK (p-ERK), two critical distal events of the RAF-MEK-ERK and PI3K-AKT activation pathways. Knock down of RAGE in Panc02 and PANC-1 cells significantly inhibited hypoxia-induced p-AKT and p-ERK (Figure 3c), suggesting that RAGE regulates activation of both the RAF-MEK-ERK and PI3K-AKT pathways under hypoxia. To determine whether the RAF-MEK-ERK and PI3K-AKT pathways are required for HIF1*α* signaling under hypoxia, we treated cells with the RAF inhibitor (e.g, RAF265), a MEK inhibitor (e.g., U0126), a PI3K inhibitor (e.g., LY294002), and an AKT inhibitor (e.g., 1,3-Dihydro-1-(1-((4-(6-phenyl-1H-imidazo(4,5-g)quinoxalin-7-yl)phenyl)methyl)-4-piperidinyl)-2H-benzimidazol-2-one trifluoroacetate salt hydrate). All of these inhibitors partly inhibited hypoxia-induced HIF1*α* expression (Figure 3d) and transactivation (Figure 3e). Moreover, a combination of MEK and AKT inhibitors completely inhibited hypoxia-induced HIF1*α* expression (Figure 3d) and transactivation (Figure 3e). In addition, the combination of MEK and AKT inhibitors inhibits the ability of RAGE overexpression to induce HIF1*α* expression (Figure 3f) and transactivation (Figure 3g) under hypoxic conditions. These findings suggest that activation of the RAGE-mediated KRAS-RAF-MEK-ERK and KRAS-PI3K-AKT pathways contributes to hypoxia-induced HIF1*α* signaling activation in pancreatic tumor cells.

### Hypoxia-induced autophagy via the RAGE-KRAS-HIF1*α* pathway is a survival mechanism in pancreatic tumor cells

**Figure 4:**
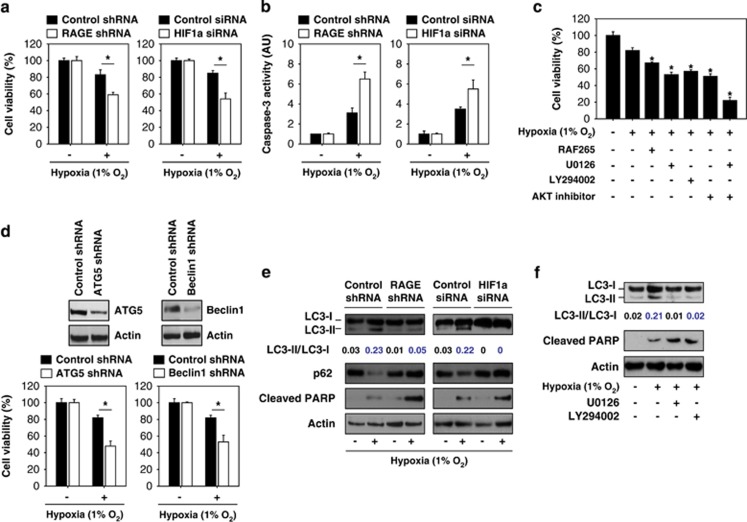
Hypoxia-induced autophagy via the RAGE-KRAS-HIF1*α* pathway is a survival mechanism in pancreatic tumor cells. (**a** and **b**) Indicated Panc02 cells were treated with 1% O_2_ for 24 h and cell viability (**a**) and caspase-3 activity (**b**) were then assayed (*n*=3, *P<0.05). (**c**) Panc02 cells were treated with 1% O_2_ for 24 h with or without potential RAF inhibitor (e.g., RAF265, 1 *μ*M), MEK inhibitor (e.g., U0126, 10 *μ*M), PI3K inhibitor (e.g., LY294002, 10 *μ*M), and AKT inhibitor (e.g., 1,3-Dihydro-1-(1-((4-(6-phenyl-1H-imidazo(4,5-g)quinoxalin-7-yl)phenyl)methyl)-4-piperidinyl)-2H-benzimidazol-2-one trifluoroacetate salt hydrate, 25 *μ*M). Cell viability was assayed (*n*=3, *P<0.05). (**d**) Panc02 cells were transfected with control shRNA, ATG5 shRNA, and Beclin1 shRNA for 48 h, and then treated with 1% O_2_ for 24 h. Cell viability was assayed (*n*=3, *P<0.05). (**e** and **f**) Indicated Panc02 cells were treated with 1% O_2_ for 24 h with or without MEK inhibitor (e.g., U0126, 10 *μ*M) and PI3K inhibitor (e.g., LY294002, 10 *μ*M). Protein levels were assayed by western blot

Although HIF1*α* has a major role in the cell survival response to hypoxia,^30,31^ it also is associated with cell death in some instances.^32^ Given that the RAGE-mediated KRAS pathway is an important regulator of HIF1*α* signaling (Figures 2 and 3), we therefore determined whether the knock down of RAGE regulates cell survival and cell death under hypoxic conditions. Similar to our previous study,^21^ knockdown of RAGE and HIF1*α* decreased cell viability as assessed in a CCK8 assay (Figure 4a) and increased apoptosis demonstrated by enhanced caspase-3 activity (Figure 4b) following hypoxia. The RAF inhibitor (e.g., RAF265), MEK inhibitor (e.g., U0126), PI3K inhibitor (e.g., LY294002), and AKT inhibitor (e.g., 1,3-Dihydro-1-(1-((4-(6-phenyl-1H-imidazo(4,5-g)quinoxalin-7-yl)phenyl)methyl)-4-piperidinyl)-2H-benzimidazol-2-one trifluoroacetate salt hydrate) all increased hypoxia-induced cell death (Figure 4c). These findings suggest that activation of the RAGE-KRAS-HIF1*α* pathway under hypoxia promotes the survival of pancreatic tumor cells. Previous studies suggested that autophagy, a lysosome-mediated degradation pathway, is a major pro-survival mechanism of various tumor cells in response to hypoxia.^33,34^ Similarly, the knock down of key autophagy regulators (ATG5 and Beclin1) by shRNA inhibits their protein expression (data not shown) and promotes pancreatic tumor cell death under hypoxic conditions (Figure 4d), supporting a pro-survival role of autophagy in the hypoxic tumor microenvironment.^35^ Importantly, the knock down of RAGE and HIF1*α* in Panc02 cells inhibits hypoxia-induced autophagy and increases hypoxia-induced apoptosis by western blot analysis of LC3-II/LC3-I ratio (a marker of autophagy) and cleaved PARP (a marker of apoptosis) (Figure 4e). The adaptor protein p62/SQSTM1 is decreased when autophagy is induced, whereas it accumulates when autophagy is impaired.^36^ Consistently, the knock down of RAGE and HIF1*α* in Panc02 cells prevents the expected decrease in p62 levels following induction of hypoxia (Figure 4e). Moreover, MEK inhibition (e.g., U0126) and PI3K inhibition (e.g., LY294002) also inhibit autophagy (LC3-II/LC3-I ratio) and increase apoptosis (cleaved PARP) in response to hypoxia (Figure 4f). Taken together, our findings suggest that autophagic flux is enhanced under hypoxic conditions, dependent on cell survival signals generated by activation of the RAGE-KRAS-HIF1*α* pathway.

### Genetic ablation of RAGE impairs mutated KRAS and HIF1*α* signaling *in vivo*

**Figure 5:**
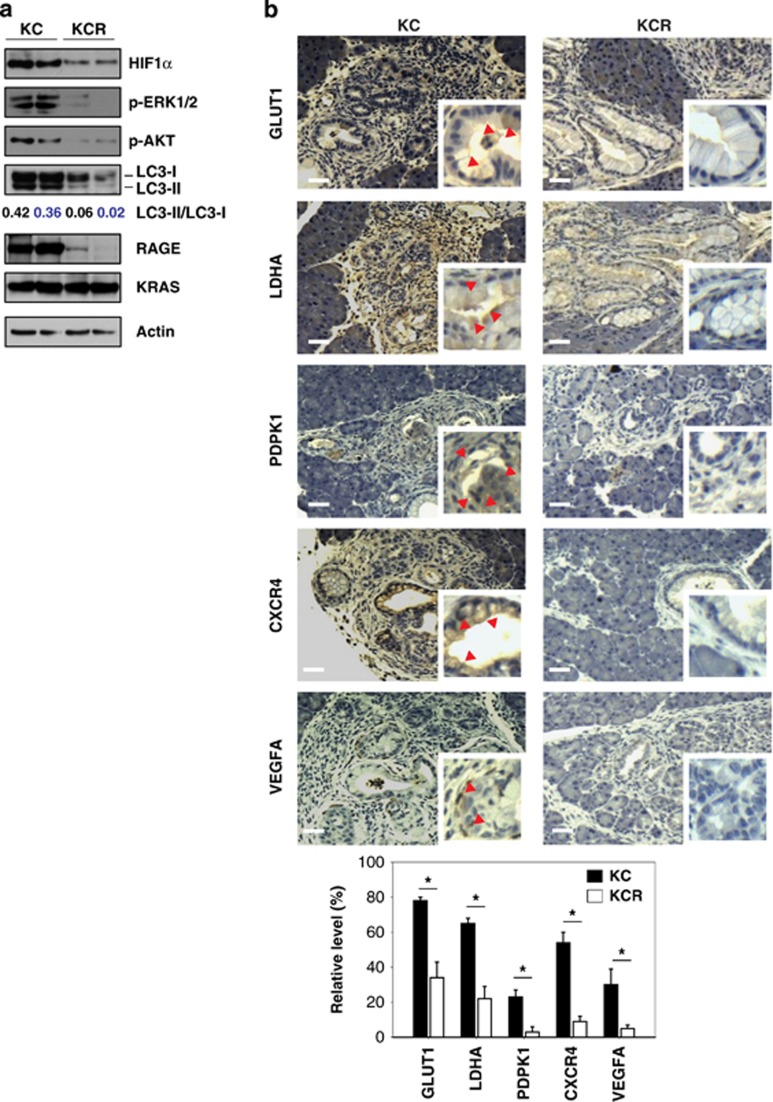
Depletion of RAGE in mice impairs KRAS-mediated HIF1*α* signaling *in vivo*. (**a**) Western blot and (**b**) immunohistochemistry analysis of indicated protein expressions from pancreatic specimens at 18 weeks of age (quantitative analysis drawn from five separate high power fields, *P<0.05). Bar=100 *μ*m. Representative positive cells were marked by red arrows

Studies of human pancreatic ductal adenocarcinoma have been greatly facilitated by the development of a genetically engineered mouse model that expresses oncogenic *KRAS* under a pancreatic promoter *Pdx1-Cre:Kras*^*G12D/+*^ (KC mice).^37^ Our published findings demonstrate that RAGE promotes KRAS^G12D^-driven pancreatic tumorigenesis.^19^ Knock out of RAGE delayed KRAS-driven pancreatic intraepithelial neoplasia progression and prolonged murine survival (KCR mice).^19^ Mutant KRAS enhances HIF1*α* expression and activity in the development of several tumors.^38^ To address the role of RAGE in the regulation of KRAS-mediated HIF1*α* hypoxia signaling, we stained HIF1*α* and its target proteins in KC and KCR mice. Compared with KC mice, KCR mice exhibited significantly downregulated expression of HIF1*α* (Figure 5a) and its targets genes involved in angiogenesis (VEGFA), anaerobic metabolism (GLUT1, LDHA, and PDPK1), and metastasis (CXCR4) (Figure 5b). Indeed, our previous studies demonstrated that depletion of RAGE in mice decreases the KRAS-mediated autophagy response and bioenergetics *in vivo*.^19,22^ Similar to our observations *in vitro* (Figures 3c and 4e), knock out of RAGE inhibits KRAS-mediated p-ERK1/2, p-AKT, and LC3-II (Figure 5a). Collectively, these findings suggest that RAGE expression is required for KRAS-mediated activation of HIF1*α* signaling and pancreatic cancer bioenergetics and growth *in vivo*.

## Discussion

Pancreatic cancer, is characterized by near-universal mutations in the KRAS oncogene and a hypoxic and pro-inflammatory tumor microenvironment. In this study, we demonstrate that NF-*κ*B- driven expression of RAGE sustains KRAS and HIF1*α* activation in response to hypoxia. This facilitates pancreatic tumor growth and proliferation. These findings provide a novel mechanistic link between NF-*κ*B, KRAS, and HIF1*α*, three potent oncogenes in the cellular response to hypoxia and pancreatic tumor development.

We have demonstrated that constitutive RAGE activation is a characteristic signature in pancreatic cancer cells.^19^ However, how RAGE is activated in the hypoxic tumor microenvironment is not fully understood. Many stressful stimuli such as inflammation, hypoxia, and oxidative stress can activate the NF-*κ*B pathway and increase RAGE expression in immune and tumor cells. Our previous study demonstrated a positive feedback loop between RAGE and NF-*κ*B during oxidative stress in pancreatic tumor cells exposed to chemotherapy.^20^ NF-*κ*B activation within the tumor microenvironment regulates a number of genes important in cell death, metabolism, immunity, and inflammatory responses, contributing to oncogenesis.^39^ HIF1*α* is a master regulator of hypoxia^15^ and its expression and activity is also controlled by NF-*κ*B.^40,41^ Here, we demonstrated that genetic and pharmacologic inhibition of NF-*κ*B, but not HIF1*α*, significantly inhibits hypoxia-induced RAGE expression. These findings suggest that NF-*κ*B is the major transcription factor regulating the expression of RAGE in response to various stressors, including hypoxia, in pancreatic tumor cells.

Although our knowledge of the molecular function of RAGE during neoplastic transformation and malignant progression is limited, recent experimental data support a direct link between RAGE activation and proliferation, survival, migration, and invasion of tumor cells.^42,43^ Blockade of the RAGE signaling pathway suppresses tumor growth, angiogenesis, and metastases.^16,17^ We and others have demonstrated that RAGE-deficient mice are resistant to chemically-induced skin carcinogenesis, colitis-associated cancer induction, as well as pancreatic tumorigenesis.^19,43, 44, 45^ However, RAGE has shown tumor suppressor activity in several cancer types, including esophageal squamous cell^46^ and oral squamous cell carcinomas,^47^ lung carcinoma,^48^ chondrosarcoma,^49^ and rhabdomyosarcoma.^50^ Thus, understanding the expression and function of RAGE in the setting of cancer is important in developing novel targeted therapies.

Our findings indicate that RAGE preferentially binds to mutant KRAS to activate the RAF-MEK-ERK and PI3K-AKT pathways, which contributes to HIF1*α* activation *in vitro* and *in vivo*. Oncogenic KRAS mutation is the key regulator in pancreatic cancer maintenance.^4,6^ Many receptors for growth factors and cytokines are able to activate KRAS either by direct or indirect protein interaction.^4,6^ Similarly, we demonstrated that hypoxia increases the interaction between RAGE and KRAS in pancreatic tumor cells. Active KRAS modulates a number of signaling pathways. The two best-studied pathways that are activated by KRAS are the RAF-MEK-ERK pathway and the PI3K-AKT pathway. The RAF-MEK-ERK pathway ultimately promotes cell proliferation and migration, whereas the PI3K-AKT pathway leads to enhanced protein synthesis and limits apoptosis. The oncogenic KRAS-mediated signaling pathway is required for HIF1*α* stabilization and activation in colon cancers.^38,51^ Our findings show that knockdown of RAGE in pancreatic tumor cells inhibits mutant KRAS activation and subsequent HIF1*α* stabilization and transactivation. Moreover, knock out of RAGE in mice prevents oncogenic KRAS-driven HIF1*α* signaling activation.

Autophagy has an important role in both physiological and pathological conditions. However, the role of autophagy in tumorigenesis depends on tumor type and stage.^52,53^ An early study indicated that autophagy promotes pancreatic cancer growth.^54^ Inhibition of autophagy by chloroquine limits pancreatic cancer growth in *in vivo* xenograft models.^54^ More recently, deletion of certain genes (e.g., ATG5 and ATG7) has been found to be involved in the core mechanism of autophagy, leading to the blocking of the progression of KRAS-driven tumor from precursor lesions into full pancreatic ductal adenocarcinoma in mice.^55,56^ This halt in progression is due to the activation of p53, a transcription factor that regulates a large number of genes and guards against genomic instability.^55^ However, a recent study shows that ATG5 deletion impairs the progression of pre-malignant PanIN to invasive pancreatic ductal adenocarcinoma in the setting of p53 loss and that p53 status does not impact response to autophagy inhibition.^56^ These results highlight the complex relationship between autophagy and p53 in the pancreatic cancer. Our previous study demonstrated that RAGE-mediated autophagy is required for STAT3 signaling activation during pancreatic tumorigenesis.^19^ Consistent with our results in mice, hypoxia-induced autophagy via the RAGE-KRAS-HIF1*α* pathway is a survival mechanism in pancreatic tumor cells.

**Figure 6:**
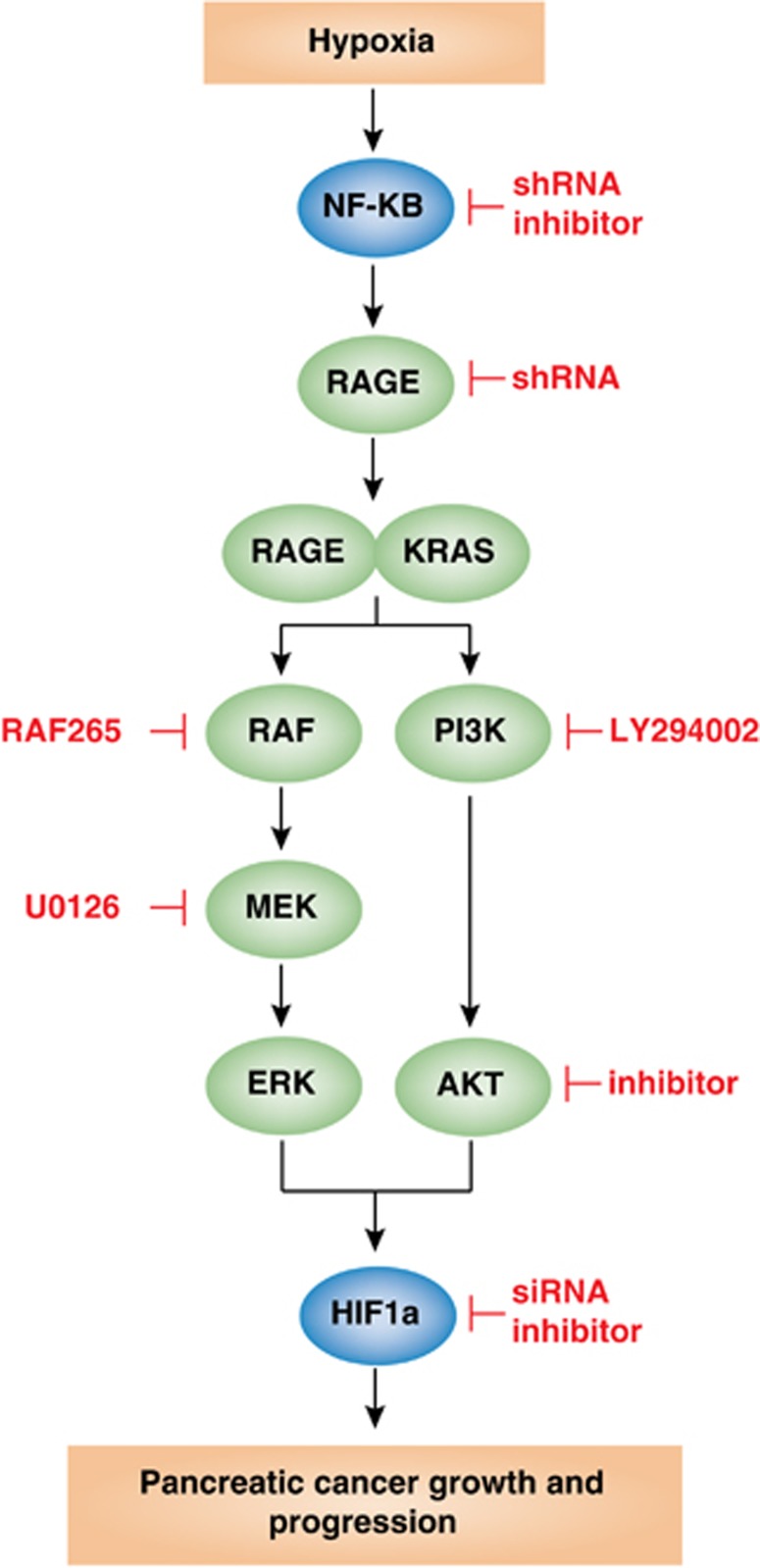
Hypoxia promotes pancreatic cancer initiation, progression, and metastasis through the activation of NF-*κ*B-RAGE-KRAS-HIF1*α* pathway

In summary, this study demonstrates that RAGE expression is regulated by a NF-*κ*B-dependent pathway and the upregulated binding of RAGE to mutant KRAS facilitates HIF1*α* activation in a range of pancreatic tumor cells, as well as a spontaneous pancreatic tumor model in transgenic mice (Figure 6). Our study extends our previous work by showing that expression of RAGE is involved in inflammation, metabolism, and autophagy changes in pancreatic cancer growth and progression.^18, 19, 20, 21, 22^ Preferential binding of RAGE to mutant KRAS also suggest that acquisition of this mutation confers a survival advantage to early precursor and tumor cells in the emergent tumor microenvironment. Lastly, these findings suggest that the inhibition of RAGE, especially when combined with more classic chemotherapeutic agents, could be a promising therapeutic strategy in patients with pancreatic cancer.

## Materials and Methods

### Reagents

The antibodies to cleaved PARP, ATG5, Beclin1, NF-*κ*B p65, LDHA, cyclinD1, VEGFA, p-AKT, AKT, p-ERK1/2, actin, and ERK1/2 were obtained from Cell Signal Technology (Boston, MA, USA). The antibodies to RAGE, BNIP3, Lamin A, HIF1*α*, PDPK1, GLUT1, and CXCR4 were obtained from Abcam (Cambridge, MA, USA). The antibodies to actin, tubulin, and BNIP3L/NIX were obtained from Sigma (St. Louis, MO, USA). The antibodies to LC3 and HMGB1 were obtained from Novus (Littleton, CO, USA). The antibodies to KRAS and p62 were obtained from Santa Cruz (Santa Cruz, CA, USA). The nuclear and cytoplasmic extraction kit was obtained from Pierce (Rockford, IL, USA). HIF1*α* inhibitor (methyl 3-((2-(4-(2-adamantyl)phenoxy)acetyl)amino)-4-hydroxybenzoate) was obtained from Santa Cruz. Other agents and inhibitors were obtained from Sigma or Selleck Chemicals (Houston, TX, USA).

### Cell culture

Pancreatic tumor cell lines were obtained from ATCC and NIH. All cell lines were cultured in RPMI or DMEM medium 1640 supplemented with 10% heat-inactivated fetal bovine serum, 2 mM glutamine, and antibiotic-antimycotic mix in a humidified incubator with 5% CO_2_ and 95% air. For hypoxia treatment, petridishes containing cells were incubated in a hypoxic chamber (Forma Scientific, Marietta, OH, USA) with a 94:5:1 mixture of N_2_/CO_2_/O_2_ as previously described.^14^

### Mouse strains

RAGE-knockout (*Rage*^*−/−*^) mice (SVEV129 × C57BL/6) were a kind gift from Dr. Angelica Bierhaus.^57^ *Pdx-1-Cre* and *Kras*^*G12D/+*^ transgenic mice on C57BL/6 background were received from the MMHCC/NCI Mouse Repository. The genotypes *Pdx1-Cre:Kras*^*G12D/+*^ (termed KC mice) and *Rage*^*−/−*^ were crossed to generate conditional-mutant mice (*Pdx1-Cre:Kras*^*G12D/+*^: *Rage*^*−/−*^) termed KCR mice. Genotyping was performed by standard polymerase chain reaction as described in our previous study.^19^ Pancreatic tissues were collected from age- and sex-matched KC and KCR mice at several different time points and fixed immediately in 10% neutral buffered formalin.

### Cell viability assay

Cell viability was evaluated using the Cell Counting Kit-8 (CCK-8) (Dojindo Laboratories, Tokyo, Japan) according to the manufacturer's instructions.

### RNA interference and gene transfection

RAGE short hairpin RNA (shRNA), ATG5 shRNA, Beclin1 shRNA, p65 shRNA, and control shRNA were obtained from Sigma, whereas pUNO1-RAGE cDNA was obtained from InvivoGene (San Diego, CA, USA). These shRNAs or cDNA were transfected into cells using the Lipofectamine 2000 reagent (Life Technologies, Carlsbad, CA, USA) according to the manufacturer's instructions. To generate stable shRNA-expressing lines, positive cells were selected with 1–2 *μ*g/ml puromycin for two to three weeks. HIF1*α*- small interfering RNA (siRNA) and control siRNA from Santa Cruz Technology were transfected into cells using X-tremeGENE siRNA reagent (Roche Applied Science, Stockholm, Sweden) according to the manufacturer's instructions.

### Western blotting

Protein lysates from cells were electrophoresed on 4–12% Criterion XT Bis-Tris gradient gels (Bio-Rad, Philadelphia, PA, USA) and transferred to a nitrocellulose membrane (Bio-Rad). After blocking with 5% milk, the membrane was incubated for 3 h at room temperature or overnight at 4 °C with various primary antibodies (PARP (1 : 1000), ATG5 (1 : 1000), Beclin1 (1 : 1000), NF-*κ*B p65 (1 : 500), LDHA (1 : 1000), cyclinD1 (1 : 1000), VEGFA (1 : 5000), p-AKT (1 : 1000), AKT (1 : 1000), p-ERK1/2 (1 : 1000), ERK1/2 (1 : 1000), RAGE (1 : 500), BNIP3 (1 : 500), Lamin A (1 : 1000), HIF1*α* (1 : 500), PDPK1 (1 : 1000), BNIP3L/NIX (1 : 500), LC3 (1 : 1000), HMGB1 (1 : 2000), KRAS (1 : 100), and p62 (1 : 500)) in 5% milk. After incubation with peroxidase-conjugated secondary antibodies in 5% milk for 1–2 h at room temperature, protein bands were visualized by enhanced chemiluminescence (Pierce) and then quantified using the Gel-pro Analyzer software (Media Cybernetics, Bethesda, MD, USA). The diagram shows quantitation relative to actin.

### Immunofluorescence analysis

Cells were cultured on glass cover-slips and fixed in 4% formaldehyde for 20 min at room temperature before detergent extraction with 0.1% Triton X-100 for 10 min at room temperature. Cover slips were saturated with 2% bovine serum albumin (BSA) in phosphate buffered saline (PBS) for 1 h at room temperature and processed for immunofluorescence with primary antibodies followed by secondary Alexa Fluor 488 or Cy3-conjugated IgG (Invitrogen, San Diego, CA, USA), respectively. Nuclear morphology was visualized with the use of Hoechst 33342 (Invitrogen) stain.

### Immunohistochemistry analysis

Paraffin embedded specimens were deparaffinized in xylene, subjected to heat mediated antigen-retrieval in 10 mM sodium citrate (pH 6.0), permeabilized in 0.2% Triton-100 (Sigma) and treated with 100–400 *μ*l blocking solution (Cell Signal Technology). After removal of blocking solution, tissue sections were incubated with 100–400 *μ*l primary antibody as indicated in Figure 5 overnight at 4 °C followed by horseradish peroxidase-conjugated secondary antibody (Cell Signal Technology). The signal was amplified with SignalStain Boost Detection Reagent (Cell Signal Technology) and detected using SignalStain DAB reagent (Cell Signal Technology).

### Immunoprecipitation analysis

Cells were lysed at 4 °C in ice-cold modified radioimmunoprecipitation (RIPA) lysis buffer (Millipore, Billerica, MA, USA) and cell lysates were cleared by centrifugation (12 000 *g*, 10 min). Concentrations of proteins in the supernatant were determined by bicinchoninic acid assay. Before immunoprecipitation, samples containing equal amount of proteins were pre-cleared with protein A or G agarose/sepharose (4 °C, 3 h) and subsequently incubated with various irrelevant IgG or specific antibodies in the presence of protein A or G agarose/sepharose beads for 2 h or overnight at 4 °C with gentle shaking. Following incubation, agarose/sepharose beads were washed extensively with PBS and the proteins were eluted by boiling in 2 × sodium dodecyl sulfates (SDS) sample buffer before SDS-PAGE electrophoresis.

### NF-*κ*B and HIF1*α* transcription activation assay

The transcriptional activation of NF-*κ*B and HIF1*α* were assayed by TransAM NF-*κ*B p65 (#40096) and HIF1*α* (#47096) Transcription Factor ELISA Kits (Activemotif, Carlsbad, CA, USA) according to the manufacturer's instructions.

### Caspase-3 activity assay

Caspase-3 activity assay was performed using Caspase-3 Colorimetric Assay Kit from Calbiochem (San Diego, CA, USA) according to the manufacturer's instructions.

### Statistical analysis

Data are expressed as mean±S.D. of three independent experiments. One-way ANOVA was used for comparison among the different groups. When the ANOVA was significant, *post hoc* testing of differences between groups was performed using an LSD test. A *P*-value<0.05 was considered significant.

## Abbreviations

AP: ammonium pyrrolidinedithiocarbamate
Bay: (and bay 11-7082)
BNIP3: BCL2/adenovirus E1B 19 kDa interacting protein 3
CoCl_2_: cobalt chloride 2
HIF1: hypoxia-inducible factor 1
HMGB1: high mobility group box 1
PDPK1: 3-phosphoinositide dependent protein kinase 1
LDHA: lactate dehydrogenase A
RAGE: the receptor for advanced glycation end products
VEGFA: vascular endothelial growth factor A
